# Integrated laboratory classes to learn physiology in a psychology degree: impact on student learning and experience

**DOI:** 10.3389/fpsyg.2023.1266338

**Published:** 2023-11-13

**Authors:** Judit Sánchez, Beatriz Navarro-Galve, Marta Lesmes, Margarita Rubio, Beatriz Gal

**Affiliations:** ^1^Departamento de Educación y de la Comunicación, Universidad Europea de Madrid, Madrid, Spain; ^2^Facultad de Ciencias Biomédicas y de la Salud, Universidad Europea de Madrid, Madrid, Spain; ^3^Departamento de Medicina, Facultad de Ciencias Biomédicas y de la Salud, Universidad Europea de Madrid, Madrid, Spain; ^4^Facultad de Ciencias de la Salud, Camilo José Cela University, Madrid, Spain; ^5^Departamento de Ciencias Médicas Básicas, Facultad de Medicina, Universidad CEU San Pablo, Madrid, Spain

**Keywords:** physiology teaching, PBL methodology, clinical cases, laboratory classes, integrated laboratory classes, academic performance

## Abstract

Physiology is a fundamental discipline to be studied in most Health Science studies including Psychology. Physiology content is perceived by students as rather difficult, who may lack vision on how to relate it with their professional training. Therefore, identifying novel active and more engaging pedagogical strategies for teaching physiology to psychology students may help to fill this gap. In this pilot study, we used the PBL methodology developed around a clinical case to evaluate psychology students’ experience and learning in two laboratory classes modalities. The aim of this study was to compare the undergraduates’ preference for laboratory classes taught either independently (cohort 1, *n* = 87 students) or integrated into the PBL-oriented clinical case (cohort 2, *n* = 92 students) for which laboratory classes were transformed into Integrated Laboratory Classes (ILCs). The students’ academic performance was also evaluated to look for quantitative differences between cohorts. We found similar overall academic scores for the Physiology course between cohorts. Interestingly, when we compared the academic scores obtained in the theoretical content from each cohort, we found a significant improvement (*p* < 0.05) in cohort 2 where the students achieved better results as compared to cohort 1. A subset of students was asked to fill a questionnaire assessment on their experience and found that 78.9% of them preferred integrated laboratory classes over laboratory classes alone. They consistently reported a better understanding of the theoretical content and the value they gave to ILCs for learning. In conclusion, our pilot study suggests that integrating laboratory classes into PBL-oriented clinical contexts help to retain core physiology contents and it can be considered as an engaging learning activity worth implementing in Psychology teaching.

## 1. Introduction

Physiology is considered a challenging discipline in most Health Science studies. Psychology students in particular usually consider physiology as a less relevant part of their professional training even though it provides an essential scientific understanding of the biological processes underlying human behavior and cognition (Vanags et al., 2012; Lloyd et al., 2019). Understanding physiology entails successful comprehension, integration and application of several complex concepts (Rehan et al., 2016). The very nature of the discipline, the way it is taught and the student’s preconceptions have all been described to contribute to this perceived intrinsic difficulty (Michael, 2007; Slominski et al., 2019). Finding new pedagogical strategies for teaching physiology to psychology students is thus important to enhance their understanding of the biological underpinnings of human behavior and to foster interdisciplinary connections within the Health Science field.

The ability to make predictions about how external and internal changes affect the state of a biological system is central to physiology (Zohar, 1995). Active methodologies are defined by educational researchers as any activity that ‘involves students in doing things and thinking about the things they are doing’ by engaging them cognitively and meaningfully with the materials (Bodemer and Ploetzner, 2004; Goodman et al., 2018), leading to a better understanding of complex ideas and mastering difficult skills (Chi, 2009). Research suggests that active teaching methodologies with multiple and interactive external interactions are required for effective students’ engaging students (Bodemer and Ploetzner, 1998). It has been discussed that those who actively relate sources of information are able to create mental representations that are more coherent when it comes to content application than those who do not make these connections (Cakir, 2008; Anwar, 2019). Under this perspective, for learning to be effective, students must develop the capacity to evaluate and use information. New concepts that fit within preexisting mental structures are better retained and understood.

All this suggest that students would be better able to handle with difficulties if they actively engage in the cognitive processes required to build connections amongst separate information pieces (Hopper, 2016). This will not only help in their learning but for physiology teaching in particular, it may be an opportunity for them to handle with difficulties in understanding. Anwar states that in active learning: “students’ participation differs from more traditional learning environments in their level of participation and collaboration.” Hopper (2016) emphasizes that “the more students participate in activities that are designed to improve learning and competence development (such writing, evaluating, synthetizing, analyzing, and thinking) the more competent they will become.” Thus, active learning pedagogies help to develop and assess mental models which need to be used in order to understand newly introduced concepts (Graffam, 2007). This in turn enables students to generate new ideas which go beyond the learning material (Chi and Wylie, 2014).

In recent decades, the importance of teaching basic sciences such as Physiology in an integrated manner has been emphasized (Sánchez et al., 2020; Tun et al., 2020). This advocates for a clinical perspective to be included from the initial formative years to better equip students with the ability to navigate the complexities of psychology. This is especially true for laboratory classes which represent a major step in most Health Science curricula. Different methodologies such as activity-based learning (ABL) and problem-based learning (PBL) have been previously used in integrated curricula. PBL is a methodology in which the starting point is a problem that enables students to develop a hypothesis and identify their own learning needs. PBL is typically taught using small groups (usually around 8–10 students) with a tutor who guide students to keep on track of learning objectives of the task (Trullàs et al., 2022). PBL methodology obtained a high level of satisfaction, especially among students (Trullàs et al., 2022).

Using PBL methodologies can thus help to better engage Psychology students in learning physiology concepts, especially in relation to more real case scenarios. Integration of laboratory classes into the PBL framework has been shown to significantly improve understanding, leading to the development of critical thinking and other major competences (Matsuo et al., 2011; Azer et al., 2013). By providing coherence and contextualization of the laboratory classes, this approach enable students to gain a deeper understanding of the content (Hammerness, 2006; Canrinus et al., 2019). Based on this approach, we have recently introduced workstation learning activities (WSLA) as an active methodology where the students organized in groups of 5–6 students rotate across different stations to work in an integrated manner the basic scientific aspects of a particular clinical case (González-Soltero et al., 2017). We demonstrated its effectiveness in more interactive and constructive knowledge for medical students (Sánchez et al., 2022). Such a framework may be particularly useful to teach practical aspects of Physiology to psychology students, as well.

Here, we investigate the effectiveness of integrating Physiology teaching with PBL-based laboratory classes by evaluating the impact on academic performance and students’ perception in the first year of the Psychology undergraduate program. Our hypothesis is that students in which the laboratory classes are contextualized into PBL develop a better knowledge as compared to those who did not have the experience. To address this hypothesis, we developed a pilot study using a PBL-oriented clinical case on Parkinson’s disease using WSLA as an example. We compare a first group (academic year 2018–2019) in which laboratory classes were taught separately from the clinical case with an experimental group (year 2019–2020) for which laboratory classes were contextualized into the PBL and transformed into Integrated Laboratory Classes (ILCs) (Azer et al., 2013). Our study demonstrates the value of ILCs for learning, and highlight the strategies used by students for knowledge integration of theoretical concepts.

## 2. Materials and methods

### 2.1. Description of the experimental cohorts

In this pilot study, we aimed to assess the impact of academic performance and student’s experience of teaching laboratory classes contextualized within the PBL applied to Physiology teaching in the first-year degree of Psychology. Two cohorts from consecutive academic years were selected: cohort 1 (*n* = 87) from 2018/19, which was considered the control group, and cohort (*n* = 92) during 2019/2020 as the experimental group. Cohort 1 was organized in three homogeneous group of students each of 25–30 students. Cohort 2 was organized in four heterogeneous group of students, two of them composed by 30 students and the other two of less than 30 students. The students of both cohorts aged between 18 and 25 years. In both cohorts there were similar distribution per gender, with 69–77% women and 23–31% men.

The methodology used to teach the subject’s content consisted in lectures (theory block), laboratory classes (practical block), and problem-based learning (PBL block). The PBL block was taught through a clinical case that integrates different learning activities using WSLA. The theoretical block was delivered in the same way in both cohorts. The main difference between cohort 1 and cohort 2, was that in the first one (2018/19), the laboratory classes were conducted independently of the PBL block. As for the second cohort (2019/20), the practices were contextualized with PBL as an additional workstation and named Integrated Laboratory Classes (ILCs) in order to provide a meaningful context.

Five clinical cases were completed in PBL blocks throughout the course. The cases consisted of a clinical scenario, a description of the patients, and various situations that students must solve in groups. Students had to rotate through different workstation learning activities. In each workstation, they worked with different aspects of the clinical case through learning activities such as bibliographic research, audiovisual material production, visiting external institutions, etc.

**FIGURE 1:**
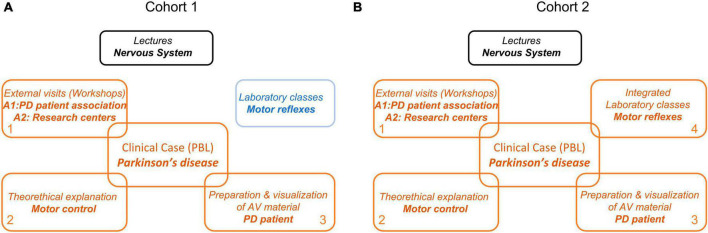
Experimental design. Tow cohorts of Psychology students were considered in consecutive academic years. Both cohorts received traditional lectures in a theory block (black box). **(A)** Cohort 1 (2018/19) experienced laboratory classes on the motor reflexes (blue) totally independent of the clinical case Parkinson’s disease (PD). The clinical case was conducted using the WSLA methodology consisting of different learning activities developed through workstations (orange). WSLA integrated external visits (workshops) to research institutions relevant to PD (workstation 1) and theoretical explanations of the clinical case in particular (workstation 2). They were also asked to prepare audiovisual material (AV) of the experience as evaluation activities (workstation 3). Students from cohort 1 experienced the laboratory practices on the motor reflex in an independent block (blue). **(B)** Instead, students of cohort 2 experienced the laboratory practices on motor reflexes integrated with the clinical case. In this cohort, laboratory classes became another workstation (workstation 4). The aim was providing students with a more holistic view of the content.

To illustrate our approach, we here describe the example associated to the clinical case named ‘Practical Case: Parkinson’s disease’ (Figure 1). After traditional lectures about the nervous system were taught (black boxes), different aspects of motor control were addressed through PBL through different workstations for both cohorts (orange boxes; Figures 1A, B).

The clinical case considered a protagonist, Peter, who suffered uncontrollable motor symptoms, such as shaking, stiffness, and difficulty with balance and coordination. He was worried and decided to talk to his nephew who was a Ph.D. student. Peter was about to visit a Parkinson’s disease association. Understanding the case required students to integrate information from different activities conducted across different workstation:

Station 1: Students performed two external visits associated activities. In activity 1, they interviewed a neuropsychologist and a Parkinson’s patient (PD), both from the PD Association of Madrid. Previously, the students had worked on designing the interview questions. In activity 2, students visited an animal research laboratory at the Brain Mapping Center of Universidad Complutense de Madrid (UCM) where microPET imaging is used for translational research. They also visited the ‘Affective Neurolinguistics and Cognition Group’ of the UCM working with electroencephalography on human experimental subjects. Students solved problems through neuroimaging analysis, based on the training they received during these visits.
Station 2: Students were exposed to theoretical explanations relevant to the clinical case.
Station 3: For evaluation, students produced audiovisual material (video recordings), integrating all the learning activities worked on in the clinical case.

In cohort 1, the motor reflexes were practiced as independent laboratory classes (blue, cohort 1, Figure 1). In the cohort 2, the laboratory classes were added as an additional workstation, resulting in ILC (station 4; Figure 1, cohort 2). Students exercised the ILCs in the physiology laboratory on motor patellar reflexes and reaction time, where they measured their own different voluntary and involuntary motor responses. This workstation was contextually integrated with the clinical case.

### 2.2. Quantitative comparison of academic performance between cohorts

To evaluate results quantitatively, academic performance was defined from the results of the exams. The type of exams and their assessment was similar for both cohorts. Exams consisted of two parts. A test with multiple choice questions (30 questions) with 4 options each, for which correct answers to each question were maximally scored and a penalty applied for incorrect answers, giving a partial score from 0 to 8 points. Exams also included two additional short-answers questions, which were scored from o to 2 points. The final score (10 points) resulted from the summation of the two parts.

For quantitative analysis, academic scores from each of the different blocks in both cohorts were compared using an inferential non-parametric statistical study. As the two independent cohorts did not follow a normal distribution, a study was conducted using the Mann-Whitney non-parametric test. Academic scores were ranked from 0 to 10.

### 2.3. Analysis of questionnaire assessment

Additionally, an observational study was conducted by intentionally selecting a group of students of the 2019/20 cohort who also run Physiology in the 2018/19 course (*n* = 19). The rationale behind this decision was to be able to inform comparisons between the two academic experiences. To this purpose, we used a survey comprised of several questions scored with a Likert scale from 1 to 5 (see Supplementary Annex 1). The questionnaires were prepared using Google Form. In a first group of questions, we aimed to evaluate the students’ perceptions of learning activities (questions 1 to 4). We then asked students what methodology they considered contributed the most to their learning (laboratory practices alone, PBL+ILC, or none of them) (question 5). In question 6, we asked how they assessed ILC experienced in the 2019/20 academic year.

We also assessed the students’ perception about how much the clinical case helped them to learn the theoretical contents (question 7). To know more about what aspects the students remembered the most we also asked them to associate a particular concept learnt in different activities with each clinical case (question 8). Concepts included academic contents (e.g., patellar reflex, evoked potential) as well as some particular characteristics of the clinical case (e.g., the names of the patients and the narrative of the case).

Internal validation was conducted by two experts who reviewed the coherence and consistency of the questionnaire. Additional validation was certified by an expert professor external to the project (Elangovan and Sundaravel, 2021). Results from the questionnaires were analyzed using descriptive statistics of the Likert scale as quantitative measures.

## 3. Results

### 3.1. Quantitative study for academic performance

We analyzed the academic results of the theoretical block, common to both cohorts. To compare performance in the two different academic experiences, we looked for differences in the scores of the PBL block (cohort 1) vs. the PBL block in which ILCs were added as a workstation (cohort 2), as well as in laboratory classes (cohort 1) vs. ILCs (cohort 2). We also assessed the overall score of the Physiology course for both cohorts.

As shown in Table 1, we found similar overall academic scores for the Physiology course between cohorts. Interestingly, when we compared the medians obtained in theory blocks from each cohort, a significant improvement (*p* < 0.05) was found in cohort 2 (, median = 4.3; *n* = 92), where the students achieved better results as compared to Cohort 1 (, median = 2.9; *n* = 87). This trend was also reflected in the scores obtained in PBL+ILCs by cohort 2 (median 6.5) vs. PBL in cohort 1 (median 5.2), although it did not reach significance (*p* = 0.18).

**TABLE 1:** Comparison academic scores (median values and ranges are given).

	Cohort 1 (C1)	Cohort 2 (C2)	*P* (*p* <0.05)
Overall score of the physiology course	4 (2.6–5.6)	4 (3–6.7)	0.47
Theory block	2.9 (1–5.3)	4.3 (3–7)	<0.001
PBL (Cohort) vs. PBL + ILCs (Cohort 2)	5.2 (3.8–8)	6.5 (5.4–7.5)	0.18
Laboratory classes (Cohort 1) vs. ILCs (Cohort 2)	7.4 (5.7–8.1)	6.5 (5.4–7.5)	0.01 (0.008)

Regarding academic results from ILCs (Cohort 2, median = 6.5), we found them significantly lower than those obtained in laboratory classes alone (Cohort 1, median = 7.4) (*p* < 0.05, Mann-Whitney *U*-test), suggesting that any improvement in understanding the relevant concepts may not be resulting from a single factor but at a more integrative level. These differences cannot be explained by external variables such as the temporality of the content explained, different teachers or type of assessment. The content was taught in the same semester (S1) and by the same teacher and even the same internship support teachers in Cohort 1 and 2. Exams in both cohorts were also similar. Moreover, as detailed in methods, both cohorts had similar profile of the students, and we did not face exceptional situations that contribute external variables.

### 3.2. Analysis of questionnaires

**FIGURE 2:**
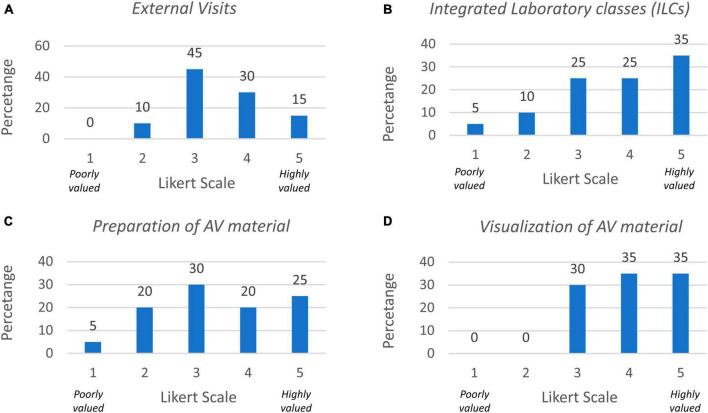
Results from questionnaires regarding learning activities in the PBL block. **(A)** Percentage of responses to questionnaires on external visits. **(B)** Integrated laboratory classes. **(C)** Preparation of AV material. **(D)** Visualization of AV material.

To better understand these differences, we next analyzed the results from the questionnaires. In general, there was variability in the answers of the questionnaires regarding how students valued the different learning activities. First, we found that while many students highly valued external visits (scoring them at 4 and 5 in the Likert scale, 45%), more than half did not score them highly (Figure 2A; mean at 3.5 of the Likert scale). Instead, most of them scored ILCs above 4 in the scales (Figure 2B; 60%). Regarding production of audiovisual material by students during the clinical case there was large dispersion in the answers with no clear preference (Figure 2C), but in general they valued their visualization with 70% of the answers above 4 (Figure 2D).

**FIGURE 3:**
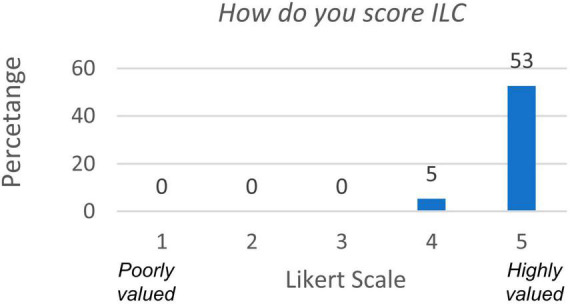
Responses to question regarding how student value ILC for their learning process.

We next assessed what methodology the students valued the most for learning and found that 78.9% of them preferred PBL+ILC over laboratory classes alone (question 5). Actually, 94.7% rated their opinion of ILCs between 4 and 5 of the Likert scale (Figure 3, question 6).

Regarding the opinion of students on how much the clinical cases helped them to learn (question 7), we found 60% rated between 4 and 5 of the Likert scale, confirming the idea that the integrated PBL block added value to learning.

Finally, we evaluated whether some concepts and ideas were able to trigger recalling of aspects of interest that were learnt over the course (question 8). With this we wanted to clarify the possible strategies used by students to build connections amongst separate information pieces. Consistent with a constructive perspective of learning, we found this to be strongly dependent on the case under study. For example, for the Clinical Case Parkinson’s disease, there was a notable higher percentage of students that answered appropriately to the questions related to the personal characteristics of the case protagonists, such as their names (73%) or the destination of their trip (68%). Regarding theoretical concepts, 56% of students correctly retrieved the clinical case in association with evoked potentials and only 25% with the concepts of neuroinflammation.

## 4. Discussion

In this work, we evaluated quantitatively if ILCs impact positively the academic performance of Physiology in first year students of psychology. Our findings show that no significant differences were obtained in the final grade for the subject. However, there was significant improvement of the score of the theoretical block of Cohort 2 students, where laboratory physiology classes were integrated and contextualized into the clinical case. Interestingly, we also found that academic results of the ILCs were significantly lower than for non-integrated laboratory classes. These results may suggest that ILCs are more challenging for students to accomplish than the non-integrated practical lesson. In spite of this difficulty, analysis of the students’ perception on questionnaires confirmed the value they give to ILCs for learning and highlight their strategies for knowledge integration of theoretical concepts.

Experimental psychologist Robert Bjork, advocates for the so-called “desirable difficulties” as learning strategies which require learners to exert an appropriate effort when learning something new. It has been demonstrated that these activities tend to result in enhanced learning (van Merriënboer et al., 2006; Bjork and Bjork, 2011; Lange and Costley, 2018; Kinsey, 2023). In curricular programs which include these planned difficulties, proper scaffolding must be embedded in the design via retrieval cues to facilitate learning the relationship between task difficulty and deep learning has been also described in the context of Health Education (Nelson and Eliasz, 2023).

Strategies to incorporate ‘desirable difficulty’ in health education include retrieval practice, spaced practice, and interleaved practice (Bjork and Bjork, 2011; Kinsey, 2023). In addition to the difficulty of the activities, it has been described that it is important to space out the day of the exam, since there are differences in remembering the concepts if the time interval until the exam is not well scheduled (Dobson, 2011). Students of cohort 1 and 2 took the exam being equally spaced and under the same conditions (structure and duration of the exam). In spite of this, the students get better results in the theoretical block having experienced ILCs. Analysis of the questionnaires confirmed this interpretation. This suggests that there was a positive impact of ILCs in spite of their difficulty, which resulted from knowledge integration at a more constructive level.

The counterintuitive idea that more difficult learning processes can enhance learning outcomes supports our results. The ILCs is a learning activity where retrieval practice is included through questions for learners to probe their knowledge. These results suggest that students learn more significantly and are able to recall better the information they learnt through ILCs. Our results add to previous reports demonstrating that retrieval practice enhances the ability to evaluate complex physiology information (Dobson et al., 2018).

In this experience we use five clinical cases each based on a clinical scenario, a description of the patients, and various situations that students must solve in groups, working in an integrated manner through different learning activities. Health Science professionals are expected to integrate content that is traditionally taught in isolation, and this may be particularly challenging in the context of psychology. When combined, active and integrated learning approaches result in an enriched learning environment which encourages students to apply knowledge to real-world scenarios and develop critical thinking skills. By doing so, students are better equipped to understand complex problems, to analyze clinical situations, and to make informed decisions in their future practice (Wood, 2003; Hmelo-Silver, 2004; Lujan and DiCarlo, 2006; Gleason et al., 2011; Parmelee et al., 2012; Thistlethwaite et al., 2012; Tayce et al., 2021; Topperzer et al., 2022). This is especially important for psychology students in order to develop their clinical competences. Our results confirm that 60% of the students consider that clinical cases helped them understand the physiology subject (question 7). Nearly all of them (94.7%) agreed that the ILCs were instrumental for understanding physiological concepts (question 6) consistent with previous experience (Azer et al., 2013).

Contextualization of the clinical case for Psychology students is crucial. All the clinical cases the students had to work with, were real. The protagonist of each case develops an illness related to the psychological and emotional domain, which naturally attracted the interest of psychology students. By feeling that the content they are studying comes close to their professional world, students gain motivation and engagement (Guilherme Guedert et al., 2022). Consistently, our results demonstrate that students better recalled relationship with the clinical cases when remembered minor details around the narrative than the academic content of the clinical case (question 8). This supports the idea that episodic and emotional cues associated to learning can influence extrinsic motivation (Schunk, 2008). Such strategies may act to stimulate health science students toward academic performance, wellbeing and satisfaction in their professional career (Leadbeatter and Gao, 2018).

As for any pilot study, there may be some potential limitation. Our study focused on a particular cohort with a limited sample. Studies in education are usually limited by factors such as the teaching load, institutional policies and logistical constrains. Future studies would include the implementation and evaluation of integrated laboratory classes in other programs and courses with a longitudinal design. Active learning methodologies seldom reach the laboratory component of basic science courses losing an important chance to provide a better learning experience for students that may be less inclined toward laboratory work.

In summary, our study supports that using active learning methodologies to teach laboratory classes within an attractive clinical context has a positive impact on learning and on the overall student’s experience. Moreover, ILCs are perceived as a motivating strategy by students which facilitates the understanding of complex physiological concepts. Using real clinical scenarios make students feel the content closer to their professional future and enhances engagement in their own learning process. This pilot study suggests that the ILCs help to retain core physiology content and it can be considered as a learning activity worth implementing in the Health curricula and in particularly in Psychology teaching.

## Data availability statement

The raw data supporting the conclusions of this article will be made available by the authors, without undue reservation.

## Ethics statement

The studies involving human participants were reviewed and approved by the Ethics Committee from the European University of Madrid (CIPI/18/060). The studies were conducted in accordance with the local legislation and institutional requirements. The participants provided their written informed consent to participate in this study.

## Author contributions

BG: Conceptualization, Investigation, Supervision, Writing – original draft, Writing – review and editing. JS: Formal analysis, Investigation, Methodology, Writing – review and editing. BN-G: Data curation, Investigation, Writing – review and editing. ML: Investigation, Methodology, Writing – review and editing. MR: Formal analysis, Investigation, Methodology, Writing – review and editing.
