# Supplementary material for: Integrated laboratory classes to learn physiology in a psychology degree: impact on student learning and experience
Source: Front Psychol. 2023 Nov 13;14:1266338. doi: 10.3389/fpsyg.2023.1266338 (PMC10681090; doi:10.3389/fpsyg.2023.1266338)
Supplement: Supplementary file 1 [file Table_1.pdf]

ANNEX 1.- QUESTIONNAIRE ASSESSMENT OF STUDENTS' EXPERIENCE

<table>
<tr><td>1</td><td>Regarding external visits, how much do you think they helped you to understand the content related to the physiology discipline?
1. meaning nothing 2. 3. 4. 5. meaning a lot</td></tr>
<tr><td>2</td><td>Regarding integrated laboratory classes, how much do you think they helped you to understand the content related to the physiology discipline?
1. meaning nothing 2. 3. 4. 5. meaning a lot</td></tr>
<tr><td>3</td><td>Regarding preparation of AV material, how much do you think they helped you to understand the content related to the physiology discipline?
1. meaning nothing 2. 3. 4. 5. meaning a lot</td></tr>
<tr><td>4</td><td>Regarding visualization of AV material, how much do you think they helped you to understand the content related to the physiology discipline?
1. meaning nothing 2. 3. 4. 5. meaning a lot</td></tr>
<tr><td>5</td><td>What methodology do you consider contributed the most to your learning?
a. Integrated laboratory classes.
b. PBL in one block and laboratory classes in another block.
c. I can't find any difference between the above options.</td></tr>
<tr><td>6</td><td>How do you assess the integration of Laboratory Classes with PBL for learning the subject (ILCs)?
1. Not interesting at all 2. 3. 4. 5. Highly interesting</td></tr>
<tr><td>7</td><td>In the Case Study on MOTOR CONTROL: What type of activity do you think helped you to learn BETTER the content related to motor control?
a. Elaboration of the video of the Practical Case
b. Evaluate the patellar reflex and reaction time in the laboratory.
c. Attend the workshop on Parkinson's disease.
d. Learning activity carried out in class on Motor Control (Basal Ganglia).
e. The explanation of the topic in the theoretical class.
f. None of the activities carried out have helped me</td></tr>
<tr><td>8</td><td>Could you relate the following terms with the corresponding Case Study?
<table>
<tr><th></th><th>Neurotoxin intoxication</th><th>Multiple Sclerosis</th><th>Parkinson Disease</th></tr>
<tr><td>Patellar reflex</td><td></td><td></td><td></td></tr>
<tr><td>Membrane potential</td><td></td><td></td><td></td></tr>
<tr><td>Evoked potential</td><td></td><td></td><td></td></tr>
<tr><td>Neuroinflammation</td><td></td><td></td><td></td></tr>
<tr><td>Pedro and Nuria</td><td></td><td></td><td></td></tr>
<tr><td>Japan travel</td><td></td><td></td><td></td></tr>
</table>
</td></tr>
</table>
